# Supplementary material for: A Likelihood Approach for Real-Time Calibration of Stochastic Compartmental Epidemic Models
Source: PLoS Comput Biol. 2017 Jan 17;13(1):e1005257. doi: 10.1371/journal.pcbi.1005257 (PMC5240920; doi:10.1371/journal.pcbi.1005257)
Supplement: S1 File — (TAR.GZ) [file pcbi.1005257.s014.tar.gz › HSPH_Online-SI-Revision/EnKF07/n100-extreme/n100-extreme_EnKF_table0.pdf]

1	R0	{2.76052, 16.3913}	{2.72045, 5.62077}	{{2.23889, 3.81438}, {3., 6.32325}}	{12.0352, 65.7088}	inf	26580.	4539.73	{518.955, 7758.79}	88.2012	1.04945	newly	10855.	6408.01	{437., 11310.}	93.6038	0.713475	Reff	0.831027	0.676804	{0.319077, 1.04701}	45.8182	ar	92090.	176912.	{159809., 186581.}	92.1078
2	R0	{2.18202, 15.1734}	{2.60542, 12.7551}	{{2.12399, 3.53801}, {3., 18.9989}}	{21.8318, 41.5859}	inf	17999.	2203.07	{168.141, 3467.22}	87.76	1.18341	newly	8027.	1448.23	{263., 4382.}	82.3669	0.999008	Reff	0.873157	0.91612	{0.378014, 2.08833}	56.251	ar	84264.	182138.	{158788., 240166.}	116.152
3	R0	{2.57891, 12.5971}	{2.33702, 6.72853}	{{0.376891, 1.52269}, {3., 18.3897}}	{30.9327, 73.1937}	inf	22711.	9349.09	{455.265, 27346.9}	88.4775	0.899296	newly	11421.	10788.3	{316., 13208.}	108.408	0.720996	Reff	0.727149	0.463336	{0.0508877, 0.815715}	51.4716	ar	90302.	185728.	{160565., 227720.}	105.675
4	R0	{2.5856, 9.28508}	{2.10961, 5.79262}	{{0.750999, 3.15349}, {3., 9.00663}}	{35.8104, 37.6137}	inf	23144.	1607.83	{380.612, 1747.86}	93.3372	1.37142	newly	15345.	1486.33	{255., 1575.}	92.144	1.26741	Reff	0.760942	0.32567	{0.0898425, 0.502353}	60.3164	ar	90312.	168975.	{162208., 180969.}	87.101
5	R0	{2.4406, 12.1341}	{2.55981, 5.44121}	{{1.53947, 3.59804}, {3., 11.6151}}	{22.5621, 55.1577}	inf	22895.	8706.5	{995.886, 12192.1}	92.2404	0.881533	newly	12782.	9891.44	{804., 38079.}	96.3782	0.666771	Reff	0.972189	0.663767	{0.309132, 0.766862}	52.0261	ar	88597.	187914.	{163795., 258803.}	112.1
6	R0	{2.72155, 15.4094}	{2.72354, 5.37995}	{{2.2443, 3.8086}, {3., 6.35829}}	{12.4089, 65.0866}	inf	26147.	5182.66	{3157.95, 10233.4}	83.0533	0.838058	newly	11271.	7190.59	{2403., 16933.}	77.2215	0.513242	Reff	0.880857	0.750338	{0.349931, 1.0609}	37.3202	ar	91682.	178206.	{160691., 188640.}	94.3743
7	R0	{2.63781, 14.3935}	{2.14378, 7.21122}	{{0.071809, 2.02599}, {3., 10.9633}}	{35.4801, 49.8995}	inf	24939.	3436.47	{194.506, 1969.21}	95.7943	1.46668	newly	11237.	2576.86	{134., 1571.}	98.7135	1.26121	Reff	0.790499	0.394549	{0.0174367, 0.67243}	59.2724	ar	91192.	172323.	{160165., 178055.}	88.9668
8	R0	{2.82391, 16.104}	{2.71514, 5.62883}	{{2.24404, 3.80888}, {3., 6.36911}}	{11.3347, 65.047}	inf	27368.	4918.62	{3089.71, 10090.6}	84.6756	0.893522	newly	11207.	6636.83	{2317., 16481.}	79.0518	0.553563	Reff	0.861801	0.736341	{0.359409, 1.05658}	37.4462	ar	92664.	177601.	{160839., 188605.}	91.6615
9	R0	{2.80645, 14.6457}	{2.70271, 4.23421}	{{2.25551, 0.187669}, {3., 6.46596}}	{11.5585, 71.0891}	inf	27606.	6473.39	{2877.18, 10183.}	84.6057	0.939103	newly	12694.	9863.31	{2238., 13797.}	90.6131	0.586507	Reff	0.94945	0.634241	{0.230903, 0.739133}	48.3231	ar	92567.	177020.	{162626., 187127.}	91.2347
10	R0	{1.86213, 5.4271}	{2.58143, 7.65558}	{{2.3179, 2.22322}, {3., 11.1215}}	{50.3359, 68.1213}	inf	10896.	6804.89	{2991.33, 9177.46}	68.7638	0.401173	newly	11765.	7265.88	{2107., 9996.}	78.5428	0.492481	Reff	0.763157	0.755656	{0.497833, 0.854389}	33.599	ar	75362.	193922.	{176475., 208903.}	157.32
11	R0	{2.27349, 14.6338}	{2.29817, 7.32063}	{{0.770413, 1.73509}, {3., 11.0233}}	{37.2615, 49.9745}	inf	19721.	3912.06	{133.433, 5864.94}	95.4905	1.35698	newly	8893.	2817.67	{111., 5659.}	93.4936	1.11509	Reff	0.854264	0.470946	{0.130071, 0.717027}	47.4452	ar	85805.	176591.	{163835., 189571.}	105.805
12	R0	{2.77261, 14.1894}	{2.5525, 6.32981}	{{1.54514, 2.88918}, {3., 8.67001}}	{18.4364, 55.3906}	inf	27138.	3254.46	{890.255, 4825.59}	92.8894	1.18149	newly	12745.	3852.93	{551., 5586.}	94.7095	0.952397	Reff	0.865526	0.413749	{0.166615, 0.636879}	54.837	ar	92508.	175624.	{166330., 181401.}	89.8474
13	R0	{2.65402, 19.3935}	{1.91096, 9.97268}	{{0.0486489, 4.11619}, {3., 14.6942}}	{41.5549, 48.5772}	inf	25765.	1553.7	{217.477, 1992.18}	93.9697	1.47959	newly	8948.	1197.53	{142., 974.}	91.6005	1.23562	Reff	0.987375	0.436404	{0.0169176, 0.719465}	63.0211	ar	91184.	166272.	{159178., 170835.}	82.3479
14	R0	{1.75297, 4.45354}	{2.54236, 5.35394}	{{1.5503, 3.56432}, {3., 11.9249}}	{50.7633, 46.8875}	inf	10727.	10394.5	{2374.39, 15180.6}	93.0374	0.404306	newly	15310.	12160.8	{1740., 40682.}	86.2402	0.500202	Reff	0.926059	0.713865	{0.134322, 0.900794}	44.9189	ar	71263.	193894.	{169382., 261499.}	172.082
15	R0	{1.99928, 7.1501}	{2.54198, 5.37628}	{{1.52559, 3.63828}, {3., 11.2326}}	{38.4494, 48.789}	inf	13996.	9290.89	{1401.56, 12639.}	86.3111	0.580995	newly	12056.	9299.04	{1028., 35057.}	81.9507	0.537903	Reff	0.80563	0.600515	{0.0612898, 0.816816}	51.4847	ar	79602.	191468.	{169259., 271168.}	140.531
16	R0	{2.67398, 10.3134}	{2.53286, 5.3313}	{{1.5182, 3.64904}, {3., 11.1058}}	{17.9808, 51.534}	inf	23578.	10321.9	{1843.09, 14532.6}	82.4986	0.684293	newly	14011.	11414.3	{1396., 35640.}	81.0273	0.495631	Reff	0.705449	0.631993	{0.147482, 1.02808}	46.7332	ar	91289.	195005.	{171434., 275610.}	113.612
17	R0	{2.44634, 16.3226}	{1.98355, 9.95778}	{{0.0262874, 4.1331}, {3., 14.6657}}	{43.3603, 38.9939}	inf	22666.	1799.97	{259.476, 2187.54}	92.3838	1.37523	newly	9419.	1420.37	{170., 1635.}	91.8655	1.22465	Reff	0.907103	0.329366	{0.010462, 0.68052}	72.0814	ar	88710.	167069.	{159829., 172070.}	88.3319
18	R0	{2.02168, 10.1676}	{2.15679, 7.23251}	{{0.181511, 2.31544}, {3., 10.8888}}	{52.3543, 35.0648}	inf	15535.	2078.93	{198.152, 1949.86}	95.4383	1.3202	newly	10392.	1540.48	{116., 1936.}	94.4719	1.24991	Reff	0.996001	0.446079	{0.0435704, 0.797361}	58.163	ar	80299.	166544.	{157558., 173433.}	107.405
19	R0	{2.89969, 15.009}	{2.69241, 4.65662}	{{2.25091, 0.131411}, {3., 6.45724}}	{11.0226, 68.9745}	inf	28996.	7110.06	{240.925, 10698.7}	86.4454	1.00028	newly	12862.	10763.1	{647., 16279.}	99.2818	0.65753	Reff	0.881738	0.67135	{0.223181, 0.929053}	44.8996	ar	93349.	179263.	{159035., 224797.}	92.0357
20	R0	{2.68933, 7.34928}	{2.39858, 5.3513}	{{1.21666, 2.31415}, {3., 7.97801}}	{24.6736, 34.254}	inf	24920.	1990.26	{603.428, 3991.36}	92.0134	1.27607	newly	20039.	3268.15	{501., 3140.}	92.2034	1.19793	Reff	0.765733	0.473816	{0.0556686, 0.738378}	51.1796	ar	91574.	182353.	{172100., 204642.}	99.1321
21	R0	{2.15352, 9.28074}	{2.5604, 5.40418}	{{1.53008, 3.62099}, {3., 11.4327}}	{32.1589, 51.5087}	inf	17876.	8990.26	{1426.55, 12168.5}	90.617	0.735885	newly	12361.	10181.4	{926., 33492.}	93.759	0.61214	Reff	0.8829	0.656832	{0.2945, 0.79698}	49.2587	ar	83845.	190013.	{166639., 264420.}	126.624
22	R0	{2.21953, 9.04387}	{2.41764, 11.1855}	{{1.94916, 4.12189}, {3., 15.9034}}	{31.797, 52.6127}	inf	18399.	4682.4	{1874.32, 9326.07}	75.6524	0.705625	newly	13126.	4535.06	{1017., 5508.}	90.2578	0.806372	Reff	0.851523	0.67763	{0.456201, 0.97418}	41.1052	ar	84856.	184746.	{171371., 189531.}	117.717
23	R0	{2.21987, 14.5347}	{2.60353, 12.8092}	{{2.12429, 3.62415}, {3., 19.0399}}	{21.3746, 43.7449}	inf	18816.	2222.16	{689.191, 3750.26}	88.19	1.07386	newly	8717.	1456.68	{405., 1540.}	84.9987	0.946087	Reff	0.868968	0.970846	{0.499323, 2.12427}	44.6134	ar	85067.	182911.	{161481., 245698.}	115.02
24	R0	{2.84268, 17.9659}	{2.64937, 12.7363}	{{2.12117, 3.46039}, {3., 18.5723}}	{13.4415, 32.2191}	inf	27580.	2617.83	{261.949, 3696.8}	93.4175	1.31519	newly	10377.	1530.25	{213., 2883.}	89.6512	1.10888	Reff	0.920602	0.862266	{0.410524, 2.12115}	43.8258	ar	92836.	181947.	{165568., 248088.}	95.9875
25	R0	{2.52706, 10.3679}	{2.52845, 6.32241}	{{1.32288, 2.73953}, {3., 8.66442}}	{23.9002, 39.0194}	inf	22248.	3527.19	{814.502, 5936.26}	91.023	1.0679	newly	13481.	4139.76	{506., 7002.}	93.6171	0.946346	Reff	0.761075	0.421243	{0.146849, 0.686229}	48.2142	ar	89623.	173818.	{163980., 180857.}	93.9431
26	R0	{2.62835, 19.1088}	{2.06831, 9.85988}	{{0.0194254, 4.14889}, {3., 14.6925}}	{36.5789, 48.4013}	inf	24689.	1709.46	{151.219, 2251.71}	93.1152	1.47827	newly	8698.	1361.68	{107., 1027.}	93.5613	1.25885	Reff	0.928833	0.333545	{0.0073579, 0.738643}	72.3066	ar	90908.	166378.	{158747., 170341.}	83.0175
27	R0	{2.76831, 19.4155}	{2.2131, 6.76474}	{{0.771908, 1.62001}, {3., 11.039}}	{29.7646, 65.158}	inf	25708.	3859.07	{180.295, 5204.15}	94.1607	1.41115	newly	8776.	2984.01	{135., 6307.}	93.084	1.06556	Reff	0.75287	0.382732	{0.0506, 0.652066}	49.6524	ar	92175.	172908.	{160846., 181806.}	87.5865
28	R0	{2.96609, 16.8903}	{2.55977, 5.52277}	{{1.52478, 3.65645}, {3., 11.1425}}	{14.9915, 67.3021}	inf	28896.	7834.14	{1143.95, 11212.4}	88.3436	0.9232	newly	11093.	7687.09	{849., 19059.}	80.566	0.573478	Reff	0.743925	0.580514	{0.116135, 0.798911}	46.5022	ar	94024.	187064.	{167730., 233554.}	98.9539
29	R0	{2.17046, 14.39}	{2.65186, 13.1958}	{{2.1327, 3.68431}, {3., 19.0094}}	{23.6838, 42.8808}	inf	18683.	2422.64	{621.465, 3741.73}	87.0329	1.02679	newly	8778.	1464.91	{468., 3117.}	84.1081	0.93402	Reff	0.946993	0.920483	{0.485209, 2.13268}	43.0721	ar	84001.	179633.	{160986., 244366.}	113.846
30	R0	{2.88772, 12.4647}	{2.06918, 5.99886}	{{0.80928, 3.27725}, {3., 9.25302}}	{32.2339, 51.8732}	inf	28167.	1356.25	{311.496, 1603.87}	95.185	1.48166	newly	14527.	1207.59	{300., 1529.}	91.6873	1.27359	Reff	0.836804	0.313206	{0.091629, 0.486885}	64.9194	ar	93273.	170470.	{164115., 183077.}	82.7645
31	R0	{2.46831, 8.14308}	{2.17073, 4.8839}	{{1.59773, 3.94713}, {3., 6.67495}}	{33.5969, 40.0239}	inf	22336.	3569.32	{229.401, 7439.27}	89.8137	1.23226	newly	17282.	3746.47	{201., 7900.}	89.7944	1.16416	Reff	0.883087	0.340703	{0.0343668, 0.638966}	65.2065	ar	88762.	180237.	{165060., 229942.}	105.7
32	R0	{1.93236, 7.38987}	{2.33569, 11.2876}	{{0.110947, 4.17807}, {3., 16.0573}}	{45.6894, 70.9361}	inf	13853.	4165.63	{1573.03, 8434.86}	73.678	0.663847	newly	12032.	3749.52	{788., 4315.}	90.298	0.852318	Reff	0.884615	0.606396	{0.0877832, 0.746549}	45.3085	ar	77654.	180547.	{169033., 185180.}	132.502
33	R0	{2.82251, 17.6954}	{2.33879, 6.84053}	{{0.426502, 1.34567}, {3., 18.9122}}	{25.4386, 65.2944}	inf	27972.	8487.15	{312.842, 20996.}	84.1099	0.987883	newly	10722.	10454.2	{385., 18555.}	108.13	0.719639	Reff	1.00304	0.463482	{0.0602784, 0.901102}	59.5272	ar	92745.	182098.	{158904., 226268.}	96.3431
34	R0	{1.87083, 12.3994}	{2.65958, 13.0683}	{{2.14852, 3.63902}, {3., 19.0586}}	{42.1604, 51.8184}	inf	12842.	1961.56	{308.771, 2366.87}	86.7735	1.0552	newly	7135.	1398.24	{128., 2208.}	86.2402	1.04274	Reff	1.01449	0.853619	{0.424634, 2.1485}	50.0444	ar	76282.	178706.	{161714., 243696.}	134.27
35	R0	{2.95779, 16.3003}	{2.26242, 7.04048}	{{0.423185, 1.34525}, {3., 19.1329}}	{25.308, 67.2173}	inf	29124.	6057.57	{398.903, 6657.47}	86.6117	1.13071	newly	12158.	7326.04	{335., 12483.}	91.9613	0.841656	Reff	1.01659	0.460214	{0.0613455, 0.920764}	60.0319	ar	93873.	177234.	{159135., 226965.}	88.8017
36	R0	{2.18718, 14.261}	{2.59453, 6.36507}	{{1.72842, 3.27745}, {3., 8.58666}}	{28.6963, 55.3673}	inf	18672.	4030.6	{1126.73, 6133.41}	89.3898	0.944666	newly	8858.	4034.61	{651., 7346.}	86.603	0.729156	Reff	0.915641	0.407074	{0.150333, 0.6684}	59.0969	ar	84379.	177011.	{164959., 184436.}	109.781
37	R0	{2.44912, 9.31118}	{2.56126, 7.47545}	{{0.789528, 2.16245}, {3., 11.2755}}	{21.5129, 38.2711}	inf	21427.	5998.36	{3113.43, 8883.79}	74.8929	0.649349	newly	14344.	6890.46	{2050., 10391.}	75.6831	0.554326	Reff	0.796601	0.738807	{0.40202, 0.858092}	32.9393	ar	88442.	191831.	{176777., 209377.}	116.9
38	R0	{2.05949, 5.72514}	{2.54305, 7.26599}	{{0.828351, 2.32146}, {3., 11.2738}}	{38.2281, 58.9039}	inf	16011.	7743.17	{4161.84, 10514.9}	64.9339	0.442627	newly	16782.	9235.39	{2845., 12527.}	75.9398	0.514182	Reff	0.827297	0.791211	{0.278202, 0.884352}	36.2414	ar	81587.	196897.	{178481., 214212.}	141.334
39	R0	{1.75505, 8.52404}	{2.2501, 7.27789}	{{0.622706, 2.23514}, {3., 10.9885}}	{61.1713, 40.9652}	inf	10952.	2684.82	{120.024, 1968.75}	100.252	1.25846	newly	8590.	1903.5	{121., 1922.}	97.5595	1.25693	Reff	0.957748	0.47475	{0.134074, 0.768122}	53.5768	ar	70925.	168533.	{158313., 175661.}	137.622
40	R0	{2.24724, 13.7861}	{2.61843, 6.44652}	{{1.71244, 3.14817}, {3., 8.44349}}	{27.6986, 53.239}	inf	18516.	3974.05	{1049.66, 6863.64}	89.6148	1.00031	newly	8935.	4085.06	{634., 10371.}	88.9932	0.811263	Reff	0.839861	0.384342	{0.181827, 0.643095}	58.3994	ar	85257.	176495.	{164574., 186325.}	107.015
41	R0	{1.77635, 8.62406}	{2.74863, 5.46776}	{{2.24196, 3.8111}, {3., 6.2848}}	{54.7346, 36.5988}	inf	11281.	5351.02	{2237.13, 9237.31}	72.6578	0.515759	newly	8585.	7111.64	{2088., 14460.}	88.1512	0.4643	Reff	0.87725	0.670333	{0.324455, 1.04028}	44.8121	ar	72941.	180825.	{164055., 196244.}	147.906
42	R0	{1.80397, 5.4932}	{2.5639, 7.73305}	{{0.871501, 2.18826}, {3., 11.4723}}	{54.0204, 69.5177}	inf	10692.	6204.52	{2906.75, 8108.89}	71.0344	0.433601	newly	11817.	6588.46	{1771., 8921.}	78.0298	0.538356	Reff	0.8332	0.713537	{0.349731, 0.808819}	36.4449	ar	73232.	190710.	{175330., 211246.}	160.419
43	R0	{2.83096, 16.7077}	{2.01455, 10.1888}	{{0.0154877, 4.01255}, {3., 14.457}}	{35.168, 39.0176}	inf	27789.	2748.56	{433.179, 2085.75}	95.2058	1.35687	newly	11209.	1885.44	{282., 1771.}	93.2724	1.17292	Reff	0.932207	0.402255	{0.00435156, 0.948932}	68.9498	ar	92740.	171090.	{162789., 175299.}	84.484
44	R0	{2.73678, 11.6021}	{2.6914, 10.7784}	{{2.31753, 4.24344}, {3., 15.3549}}	{12.0455, 38.2687}	inf	23763.	7048.74	{3017.45, 12222.4}	74.7945	0.636704	newly	12451.	5878.46	{2050., 8264.}	80.4429	0.589406	Reff	0.638655	0.697035	{0.376287, 2.21537}	54.4687	ar	92003.	194225.	{177311., 269795.}	111.108
45	R0	{2.14235, 6.98601}	{2.51878, 5.33096}	{{1.51626, 3.65508}, {3., 11.0488}}	{31.5275, 48.1164}	inf	15339.	10175.6	{2253.47, 14236.7}	78.8995	0.52829	newly	13010.	10446.	{1372., 35074.}	72.8141	0.469691	Reff	0.718266	0.623885	{0.169811, 0.863078}	46.3874	ar	82974.	195626.	{173030., 275456.}	135.768
46	R0	{2.23409, 9.21289}	{2.55805, 5.27816}	{{1.54012, 3.58701}, {3., 11.7411}}	{29.3634, 53.1371}	inf	19256.	8645.33	{1268.9, 10674.9}	87.8777	0.713219	newly	14108.	10418.1	{953., 30171.}	88.5333	0.585432	Reff	0.983357	0.69649	{0.3227, 0.862197}	49.1135	ar	85163.	189131.	{166854., 246845.}	122.081
47	R0	{2.12258, 8.15265}	{2.55426, 7.5336}	{{0.909015, 2.1862}, {3., 11.5248}}	{34.2429, 43.9292}	inf	17054.	5663.39	{2987.12, 7981.4}	73.3919	0.593284	newly	13477.	6557.06	{1886., 9288.}	76.8625	0.569362	Reff	0.871829	0.71604	{0.317793, 0.822542}	38.3005	ar	82690.	190241.	{174805., 211664.}	130.065
48	R0	{2.71771, 10.4636}	{2.56318, 7.44011}	{{0.954802, 2.28385}, {3., 11.6264}}	{17.9428, 37.794}	inf	26340.	6863.42	{3454.44, 9097.04}	75.7295	0.691787	newly	16548.	8250.47	{2280., 10713.}	80.9634	0.583664	Reff	0.927228	0.771026	{0.262398, 0.874449}	41.2143	ar	91716.	193020.	{175998., 212669.}	110.454
49	R0	{2.44369, 6.66477}	{2.36021, 4.77637}	{{1.57775, 3.79954}, {3., 6.59802}}	{26.8674, 28.3555}	inf	18124.	3708.52	{458.575, 5170.15}	88.6867	1.06239	newly	14957.	3888.37	{388., 5655.}	90.0437	1.0185	Reff	0.592717	0.350369	{0.0514004, 0.638306}	52.9105	ar	88827.	192018.	{175183., 248849.}	116.171
50	R0	{2.77383, 17.9775}	{2.27803, 9.50218}	{{0.0169157, 4.11884}, {3., 14.5979}}	{26.6801, 47.144}	inf	25584.	2046.4	{394.022, 2795.26}	92.0516	1.31498	newly	9271.	1614.73	{263., 1551.}	91.1512	1.08661	Reff	0.747852	0.417616	{0.00882701, 0.996866}	68.9911	ar	92334.	165462.	{155653., 168378.}	79.1993
